# Epidemiologic Investigation of Two Welder’s Anthrax Cases Caused by *Bacillus cereus* Group Bacteria: Occupational Link Established by Environmental Detection

**DOI:** 10.3390/pathogens11080825

**Published:** 2022-07-23

**Authors:** Patrick Dawson, Johanna S. Salzer, Caroline A. Schrodt, Karl Feldmann, Cari B. Kolton, Jay E. Gee, Chung K. Marston, Christopher A. Gulvik, Mindy G. Elrod, Aaron Villarma, Rita M. Traxler, María E. Negrón, Kate A. Hendricks, Heather Moulton-Meissner, Laura J. Rose, Paul Byers, Kathryn Taylor, Daphne Ware, Gary A. Balsamo, Theresa Sokol, Bret Barrett, Erica Payne, Saad Zaheer, Ga On Jung, Stephen Long, Ricardo Quijano, Lindsey LeBouf, Briana O’Sullivan, Erin Swaney, James M. Antonini, Marie A. de Perio, Zachary Weiner, William A. Bower, Alex R. Hoffmaster

**Affiliations:** 1 National Center for Emerging and Zoonotic Infectious Diseases, Division of High-Consequence Pathogens and Pathology, CDC, Atlanta, GA 30329, USA; hio7@cdc.gov (J.S.S.); pgx7@cdc.gov (C.A.S.); beesleyc2@gmail.com (C.B.K.); xzg4@cdc.gov (J.E.G.); cdk5@cdc.gov (C.K.M.); ylo1@cdc.gov (C.A.G.); wzg0@cdc.gov (M.G.E.); lvn4@cdc.gov (A.V.); gna9@cdc.gov (R.M.T.); yfp3@cdc.gov (M.E.N.); kah1@cdc.gov (K.A.H.); xxd7@cdc.gov (Z.W.); wab4@cdc.gov (W.A.B.); amh9@cdc.gov (A.R.H.); 2 Epidemic Intelligence Service, CDC, Atlanta, GA 30329, USA; 3 National Institute for Occupational Safety and Health, CDC, Cincinnati, OH 45226, USA; ecz4@cdc.gov (K.F.); hgj1@cdc.gov (M.A.d.P.); 4 National Center for Emerging and Zoonotic Infectious Diseases, Division of Healthcare Quality Promotion, CDC, Atlanta, GA 30329, USA; ftw2@cdc.gov (H.M.-M.); lpierose@gmail.com (L.J.R.); 5 Mississippi State Department of Health, Jackson, MS 39216, USA; paul.byers@msdh.ms.gov (P.B.); kathryn.taylor@msdh.ms.gov (K.T.); 6 Mississippi Public Health Laboratory, Jackson, MS 39216, USA; daphne.ware@msdh.ms.gov; 7 Louisiana Department of Health, New Orleans, LA 70802, USA; gary.balsamo@la.gov (G.A.B.); theresa.sokol@la.gov (T.S.); 8 Mississippi Baptist Medical Center, Jackson, MS 39202, USA; bbarrett76@gmail.com (B.B.); erica.payne@bmhcc.org (E.P.); 9 Harris County Public Health, Houston, TX 77027, USA; saad.zaheer@phs.hctx.net (S.Z.); gaon.jung@phs.hctx.net (G.O.J.); 10 Houston Health Department, Houston, TX 77054, USA; stephen.long@houstontx.gov (S.L.); ricardo.quijano@houstontx.gov (R.Q.); lindsey.templeton@houstontx.gov (L.L.); 11 Texas Department of State Health Services, Austin, TX 78714, USA; briana.osullivan@dshs.texas.gov (B.O.); erin.swaney@dshs.texas.gov (E.S.); 12 National Institute for Occupational Safety and Health, CDC, Morgantown, WV 26505, USA; jga6@cdc.gov

**Keywords:** anthrax, anthrax toxin, *Bacillus cereus*, *Bacillus tropicus*, welding

## Abstract

*Bacillus cereus* group bacteria containing the anthrax toxin genes can cause fatal anthrax pneumonia in welders. Two welder’s anthrax cases identified in 2020 were investigated to determine the source of each patient’s exposure. Environmental sampling was performed at locations where each patient had recent exposure to soil and dust. Samples were tested for the anthrax toxin genes by real-time PCR, and culture was performed on positive samples to identify whether any environmental isolates matched the patient’s clinical isolate. A total of 185 environmental samples were collected in investigation A for patient A and 108 samples in investigation B for patient B. All samples from investigation B were real-time PCR-negative, but 14 (8%) samples from investigation A were positive, including 10 from patient A’s worksite and 4 from his work-related clothing and gear. An isolate genetically matching the one recovered from patient A was successfully cultured from a worksite soil sample. All welder’s anthrax cases should be investigated to determine the source of exposure, which may be linked to their worksite. Welding and metalworking employers should consider conducting a workplace hazard assessment and implementing controls to reduce the risk of occupationally associated illnesses including welder’s anthrax.

## 1. Introduction

In the United States, human anthrax disease is rare but remains a formidable public health threat [1,2,3]. Reported cases of anthrax in the U.S. have been primarily the cutaneous and inhalation forms. Approximately 20% of cutaneous anthrax cases and nearly all inhalation anthrax cases globally are fatal without treatment—proper diagnosis and treatment can increase survival rates to nearly 100% and 55%, respectively [4]. In the U.S., anthrax has been attributed to environmental, accidental (e.g., laboratory), or intentional (e.g., the 2001 anthrax attacks [2]) exposure to *Bacillus anthracis*—a spore-forming, gram-positive, rod-shaped, toxin-producing bacterium [1]. However, there is growing scientific recognition that other naturally occurring *Bacillus* species within the *B. cereus* group may contain anthrax toxin genes and cause cutaneous or inhalation-like anthrax disease in humans possibly through occupational exposure [5,6,7,8,9,10,11]. Recent taxonomic work has elucidated at least 16 members of the *B. cereus* group that can be pathogenic and even fatal in humans [6,12].

Prior to 2020, there were five reported cases of severe anthrax pneumonia and one reported case of cutaneous anthrax caused by *B. cereus* group bacteria that expressed anthrax toxin genes other than *B. anthracis* [5,6,7,8,9,10,11]. None of these six cases were linked to laboratory exposure. Among the five anthrax pneumonia cases, four (80%) were fatal; the one survivor experienced a prolonged hospitalization and recovery period [5,6,7,9,10,11]. All five patients with anthrax pneumonia (henceforth referred to as “welder’s anthrax”) were welders or other metalworkers who worked in Louisiana or Texas, whereas the patient with cutaneous anthrax lived in Florida and was not a metalworker [6,8].

*B. cereus* group bacteria are gram-positive facultative anaerobes that are frequently toxin-producing and exist naturally in soil and dust throughout the world [13]. Human infections with *B. cereus* group bacteria are most often associated with gastrointestinal illness but may have various other clinical presentations [13]. Although *B. cereus* group bacteria are thought to be widespread in the natural environment, it is unknown to what extent *B. cereus* group bacteria containing anthrax toxin genes (other than *B. anthracis*) exist in the environment and whether the bacteria occur naturally outside of the U.S. Gulf Coast States (Texas to Florida), where all prior cases have been detected [6]. Additionally, for reasons that remain unclear, these often-fatal anthrax pneumonia cases have only been reported among welders and other metalworkers. A likely contributing factor is that long-term exposure to welding and metalworking fumes is associated with multiple types of lung injury that may affect lung function and increase susceptibility to pulmonary infections such as fatal pneumonia [14,15,16].

In 2020, the U.S. Centers for Disease Control and Prevention (CDC) confirmed two additional cases of anthrax pneumonia (one fatal) caused by *B. cereus* group bacteria among welders [6]. In each instance, the CDC Bacterial Special Pathogens Branch was notified by the Laboratory Response Network (LRN) [17] of an equivocal *B. anthracis* polymerase chain reaction (PCR) result, which was positive for one of the three markers specific to *B. anthracis*. Both isolates were positive on the BA2 marker (indicating the presence of a homologue of the pXO1 plasmid, which encodes the anthrax toxins: protective antigen, lethal factor, and edema factor) but were negative for the other two markers, one on pXO2 (encodes the capsule) and a chromosomal marker [18]. Isolates sent to the CDC Zoonoses and Select Agent Laboratory (ZSAL) were subsequently confirmed as *B. cereus* group bacteria containing anthrax toxin genes by real-time PCR and whole genome sequencing. The first patient, patient A, was a 39-year-old male Mississippi resident who had recently worked as a welder in Louisiana. Patient A was hospitalized in late April 2020 and survived, but only after more than three months of mechanical ventilation followed by inpatient rehabilitation [6,19]. The bacterium was confirmed by ZSAL to be *B. tropicus*, a species within the *B. cereus* group, and characterized as sequence type 78 (ST-78) by multilocus sequence typing (MLST). The second patient, patient B, was a 34-year-old male who resided and worked as a welder in Texas. He presented to the hospital emergency department with altered mental status in November 2020 two days after symptom onset and died from acute respiratory failure shortly after admission [19]. ZSAL confirmed patient B’s isolate as *B. cereus*, characterized as ST-108 by MLST. The clinical course for each patient is described elsewhere [19]. CDC, in collaboration with state and local health officials, conducted an epidemiologic investigation for each case in 2020 to identify possible sources of exposure to the bacteria, characterize potential risk factors for infection, and determine whether others might be at risk for infection.

## 2. Methods

### 2.1. Epidemiologic Investigations

#### 2.1.1. Patient A

During May–June 2020, a team from CDC worked closely with health officials from the Mississippi State Department of Health, Louisiana Department of Health, and other local partners to investigate possible sources of his exposure to *B. tropicus* containing anthrax toxin genes, delineate his timeline of symptoms and timeline of soil and dust exposures in the 30 days prior to symptom onset, characterize his occupational and recreational activities, conduct environmental sampling at his worksite and residence, and determine the likelihood that others might have been exposed or be at risk for exposure.

Patient A’s medical records were reviewed, and key data were abstracted; the team conducted regular discussions with patient A’s clinical care team to gather additional clinical data. The team conducted multiple interviews with representatives from the patient’s employer, the worksite safety officer, other members of the patient’s welding crew, and the patient’s family members and friends. Interviews were guided using multiple methods including the use of standardized questionnaires, calendar-assisted recall, and review of personal notes, photographs, and text messages. Discrepant or unclear information was cross-checked across multiple interviewees to clarify dates and activities, and sometimes confirmed with date- and time-stamped text messages and photographs. The worksite questionnaire included questions about activities performed on-site, specific welding activities and associated materials/equipment, types and use of personal protective equipment (PPE), and whether any other individuals from the worksite had recently been ill.

Through these interviews, two possible exposure sites of interest were identified for environmental sampling: patient A’s worksite in Louisiana—where he had worked most of the 30 days prior to symptom onset—and his residence in Mississippi—where he had multiple significant soil and dust exposures in the 30 days prior to symptom onset.

#### 2.1.2. Patient B

In December 2020, a team from CDC collaborated with Harris County Public Health, the Houston Health Department, and the Texas Department of State Health Services to conduct a similar epidemiologic investigation. Interviews were conducted with the patient’s next of kin. No interviews were conducted with coworkers as investigators were invited to the facility when the worksite was closed. Patient B’s family members were unable to identify any other significant exposure to soil or dust in the 30 days before symptom onset besides his worksite. However, his primary mode of transportation to and from the worksite was a personal bicycle, which is inherently an outdoor activity. However, as the next of kin were not able to identify potentially significant exposures to soil or dust patient B might have while riding to work, no environmental samples were collected outside of the worksite.

### 2.2. Environmental Sample Collection and Sampling Methodology

**Figure 1:**
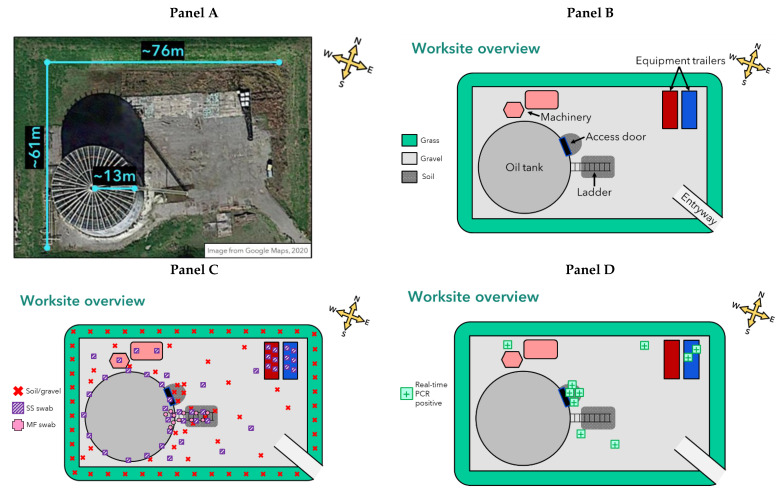
Satellite imagery of patient A’s worksite and diagram of worksite features, environmental sampling locations, and locations of samples that were real-time PCR-positive for *B. cereus* group bacteria containing anthrax toxin genes. Abbreviations: m = meter; MF = macrofoam; SS = sponge-stick. (**Panel A**): Satellite image of patient A’s worksite (Google Maps, Mountain View, CA, USA; 2020). (**Panel B**): Diagram of patient A’s worksite features and their approximate locations. (**Panel C**): Diagram of environmental sampling locations and sample type collected at patient A’s worksite. Note: All samples indicated on the equipment trailers were collected inside the trailers. (**Panel D**): Diagram of approximate locations of samples that were real-time PCR-positive for *B. cereus* group bacteria containing anthrax toxin genes at patient A’s worksite.

Detailed sampling methodologies and sampling locations for each investigation are described in Appendix A. Briefly, using a standardized sample collection protocol, surface and subsurface soil and gravel samples, sterile sponge-stick swabs, and sterile macrofoam swabs were collected from various locations within sites of interest identified in the epidemiologic investigations (patient A’s worksite (Figure 1) and residence; patient B’s worksite) [20]. Teams wore personal protective clothing and equipment as required at each worksite, plus gloves, a face mask, gowns, and shoe covers for sampling.

All samples from the investigation for patient A were stored in coolers with ice packs and wet ice and transported directly to CDC ZSAL by vehicle. Samples from the investigation for patient B were divided among three partnering laboratories: Texas State Public Health Laboratory (SPHL), Houston LRN, and CDC ZSAL. Samples were either hand-delivered to the Houston LRN and stored at 4 °C or shipped the following day to the Texas SPHL and CDC ZSAL.

### 2.3. Laboratory Testing

All samples were handled in a biological safety cabinet. Laboratory staff used a Powered Air-Purifying Respirator (PAPR) for respiratory protection. Samples were enriched, and DNA was extracted and tested by the pXO1 real-time PCR assay (an assay specific to one of the three markers for *B. anthracis*). For soil and gravel samples and macrofoam swabs, heart infusion broth was added for enrichment, and the samples were vortexed and sonicated on high for 30 s each for three cycles. The samples were then heat shocked at 65 °C for 30 min and incubated overnight at 35–37 °C. For sponge-stick swabs, the samples were first stomached at 260 rpm in 90 mL PBS + 0.02% Tween^®^ for 1 min, the eluate centrifuged at 3500× *g* for 15 min, and the resulting pellet was used for further processing [21] as above. After overnight incubation in enrichment broth, 1.0 mL was used for DNA extractions, which were performed using the EZ1 Advanced DNA Bacteria Card, EZ1 DNA Tissue kit and “Purification of DNA from Bacterial Culture Samples, Gram-positive” protocol on the EZ1 Advanced XL system (QIAGEN, Germantown, MD, USA) or manually with the QIAamp Fast DNA Stool Mini Kit (QIAGEN, Germantown, MD, USA). Manual extraction was performed when processing a low volume and/or if the EZ1 was in use for other investigations. Samples were considered positive if they had a cycle threshold (Ct) value lower than 40. A second extraction was performed on positive samples, and both extractions were required to be positive to be considered real-time PCR-positive [18,22]. Multiple PCR controls were used: an extraction control, 16s, a positive control, and a negative control. Samples with one positive and one negative extraction were considered inconclusive. Culture was attempted on all real-time PCR-positive and -inconclusive samples.

### 2.4. Whole Genome Sequencing

Whole genome sequencing was performed on patient A and B’s clinical isolates as well as the real-time PCR-positive isolate from patient A’s worksite using the Nextera FLEX kit (https://www.illumina.com; accessed on 23 October 2020) to prepare libraries that were run on an iSeq 100 instrument (Illumina, San Diego, CA, USA). The phylogeny of single nucleotide polymorphisms (SNPs) was analyzed with patient A’s clinical isolate (LA2020) and an isolate recovered from the worksite (LA2020b) and a reference panel to determine if the isolates were clonal using Parsnp in the HARVEST 1.3 suite (https://harvest.readthedocs.io/en/latest/content/parsnp.html; accessed on 23 October 2020) [23].

## 3. Results

### 3.1. Epidemiologic Investigation—Patient A

From the epidemiologic interviews, it was determined that patient A’s symptom onset was approximately 16 April 2020. He continued to work until 22 April. Patient A was hospitalized on 27 April near his residence in Mississippi. 

**Figure 2:**
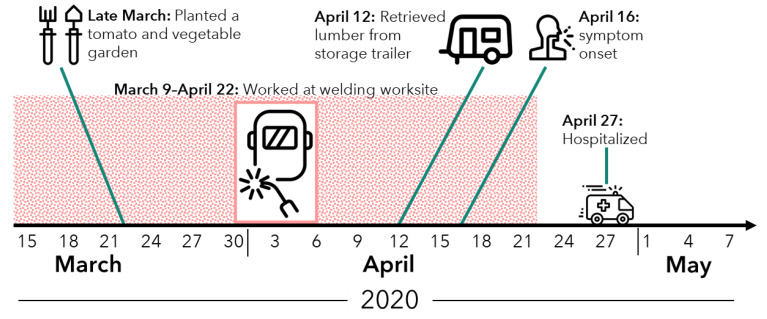
Timeline of patient A’s symptom onset, hospitalization, and significant exposures to soil and dust in the 30 days preceding symptom onset. Note: The patient worked at the welding worksite Monday through Friday each week from 9 March to 22 April 2020.

A timeline of patient A’s significant exposures to soil and dust in the 30 days before symptom onset (17 March–16 April 2020) was constructed based on employee records and interviews with family, friends, and coworkers (Figure 2). Patient A’s most prominent exposure to soil and dust was through his occupation as a welder. Since early March 2020, he had been working Monday through Friday welding a new roof onto an oil tank at an oil refinery in Louisiana. He commuted daily between his residence in Mississippi and the worksite in Louisiana by his family’s pick-up truck and occasionally a coworker’s vehicle. He often carpooled with one to two other crew members. He performed, or was near, the following worksite activities: using a wire brush to manually clean the oil tank roof, using an air compressor to blast paint off the oil tank, grinding metal near the tank, and—the hot work—performing shielded metal arc welding (SMAW; also known as stick welding) on ASTM A36 mild carbon steel rods to attach the new roof. Respiratory protection, consisting of a 3M (St. Paul, MN, USA) 6000 series half-face respirator equipped with P-100 particulate cartridges (which are designed to be effective against fumes, particles, and vapors rather than biological pathogens), was reportedly only worn during the hot work. Notably, it was remarked that patient A was the smallest member of the welding crew and therefore performed most of the work on top of the roof (e.g., wire brushing) and in tight spaces that the other crew members did not. Patient A was part of an eight-member crew that included three additional welders who were utilizing the same weld process on the same material and wore the same PPE during hot work; reportedly, no other crew members or individuals at the worksite were reported to have had any recent illnesses.

Patient A’s other significant exposures to soil and dust in the 30 days before symptom onset occurred at his residence in Mississippi: he planted a tomato and vegetable garden in the backyard in late March, spent time in a horse paddock during March and early April, and retrieved lumber from inside a mobile home being used for storage that was noted by several people present on 12 April to have black mold.

A total of 132 environmental samples were collected from patient A’s worksite in Louisiana, including 60 soil samples, 16 gravel samples, 45 sponge-stick swabs, and 11 macrofoam swabs. At patient A’s residence, a total of 53 environmental samples were collected. All 185 environmental samples were tested at ZSAL: 14 (8%) were real-time PCR-positive for the BA2 marker for the anthrax toxin genes, 167 (90%) were negative, and 4 (2%) were inconclusive as only the first extraction was positive (Table 1). Ten of the fourteen real-time PCR-positive samples were taken from patient A’s worksite (8% of all worksite samples): all four soil samples from in front of the oil tank access door, one gravel sample from each quadrant (*n* = 4 total), and two sponge-stick swabs of metal grinder tools and cabinets inside one of the equipment trailers (Figure 1). All environmental samples from patient A’s residence were negative (0%); however, 4 of the 18 samples (22%) from patient A’s work-related clothing and gear located at his residence were positive: three macrofoam swabs from one pair of his more heavily worn work boots (top and inside treads of the right boot and inside treads of the left boot) and one sponge-stick swab of the bottom of his work lunch cooler.

One (7%) of the real-time PCR-positive samples was successfully cultured: one of the soil samples taken from in front of the oil tank access door. Whole genome sequencing demonstrated that this environmental isolate was *B. tropicus* and was a genetic match to patient A’s clinical isolate (Table 2).

### 3.2. Epidemiologic Investigation—Patient B

Interviews with patient B’s next of kin and employer found that his symptoms began around 11 November 2020, and he presented to the emergency department on 13 November. He had worked as a welder at a single location for the previous 10 years and had not held any other jobs. He lived in an apartment complex, and no extracurricular metal work or other potentially significant exposures to soil or dust were identified in the 30 days before symptom onset beyond his primary worksite.

Patient B worked in a wood fabrication shop and was the only employee who welded in this building. The wood used was heat-treated pine with no chemical treatment, and the steel used was mild steel with no chemical coatings or treatments using the welding process metal inert gas (MIG). Large open doorways were the main ventilation source, and cleaning was reportedly performed with compressed air and dry sweeping. Patient B reportedly always wore a filtering face piece respirator when working and routinely wore safety glasses, gloves, ear protection, and a welding hood. Staff at the site were reportedly trained on proper PPE use for the activity, but the company did not have a formal respiratory protection program with fit testing.

In total, 108 environmental samples were collected from unique locations at patient B’s worksite: 29 soil samples, 56 sponge-stick swabs, 20 macrofoam swabs, and 3 broom bristles. Included in these counts were four swabs and one sponge sample collected from the patient’s bicycle, hung above his workstation in memoriam. None of the 108 environmental samples collected in the second investigation were real-time PCR-positive for the BA2 marker.

## 4. Discussion

The epidemiologic investigations described in this report detail the efforts undertaken to identify the source of exposure for the sixth and seventh known cases of welder’s anthrax due to *B. cereus* group bacteria in the United States [6]. The investigation of patient A’s source of exposure identified an environmental isolate at his worksite that was a genetic match to his clinical isolate—the first time that the likely source of exposure has been identified for a welder’s anthrax case. Based on the multiple real-time PCR-positive samples from that investigation, the bacteria appeared to be in soil and dust in multiple locations across the patient’s worksite and on equipment, indicating the bacteria were widespread at this occupational site, an outdoor oil tank. Work-related clothing and gear in the patient’s home were also real-time PCR-positive, which implies that the bacteria could possibly be tracked home from other locations on contaminated surfaces and materials. This may pose additional risks to workers and their household members who may come into contact with such items; although, no evidence of additional infections was found. A handful of cutaneous anthrax cases has been tied to contact with workers or their clothing [24,25,26,27].

The investigation of patient A’s source of exposure also highlights the importance of investigating all possible exposures to soil and dust in the 30 days prior to illness onset for patients experiencing welder’s anthrax. Extensive sampling conducted around his home where he had multiple other significant exposures to soil and dust prior to illness onset found no environmental evidence of *B. tropicus* with the anthrax toxin genes, which further strengthened the link to his workplace along with the finding of contaminated work clothing and gear. Since it is not known whether all patients with welder’s anthrax are exposed in an occupational setting, it is important that future investigations explore all possible locations where exposure to soil and dust occurred prior to illness onset. There are several possible reasons why the investigation of patient B’s source of exposure did not find an environmental link. Patient B’s true source of exposure may not have been his worksite. His exposure, instead, might have been somewhere along his bicycle route; the route was not sampled because no there were no known specific exposures to soil and dust. Although the bike samples were not positive, the bike might have been cleaned before it was hung. It is also possible that patient B’s worksite was the source of exposure, since a known limitation of environmental sampling is that negative results do not exclude the possibility that the organism was present. Environmental or ecological factors may have made detection more difficult or impossible, such as (1) seasonality (the environmental sampling for patient A was conducted in May–June 2020 with abundant vegetative growth around the worksite compared to December 2020 for patient B); (2) a transient exposure (the bacteria were no longer present by the time of sampling); (3) presence of inhospitable chemicals, fumes, and other environmental contaminants at patient A’s worksite, which may have favored survival of hardy *B. cereus* group bacteria by reducing ecological competition from other bacteria; (4) patient A’s worksite was primarily outdoors compared to patient B’s worksite, which was primarily indoors (albeit with some outdoor components); or (5) possible species differences in habitability between *B. tropicus* (patient A) and *B. cereus* (patient B).

Since *B. tropicus* containing anthrax toxin genes was detected in a soil sample at patient A’s worksite, and it was a genetic match to the patient’s clinical isolate, and multiple other environmental samples tested real-time PCR-positive for the anthrax toxin genes, investigators presumed the bacteria were still present at the worksite at the time of sampling and may pose a public health risk. Additionally, multiple environmental samples of work-related clothing and gear at patient A’s residence tested real-time PCR-positive for the anthrax toxin genes, indicating these items may have been contaminated with the same bacteria that caused patient A’s illness and may pose a health risk to others. The CDC National Institute for Occupational Safety and Health (NIOSH) and CDC anthrax subject matter experts worked with state health officials in Louisiana and Mississippi to develop preliminary recommendations to reduce the risk of additional exposures to and illnesses with *B. cereus* group bacteria containing the anthrax toxin genes for patient A’s worksite and family based on the findings of the investigation and applying the hierarchy of controls framework [28] (Appendix B).

In Texas, a formal NIOSH health hazard evaluation (HHE) [29] at the worksite was offered to the employer but was not accepted. A formal HHE could evaluate employees’ welding fume exposures to learn more about inhalational exposures that might increase welders’ risk of severe infection from *Bacillus* spp. and other pathogens. Serological testing of other welders was discussed for the worksites but was not pursued by the employers. Future investigations could consider the collection of serum samples from co-workers of patients with welder’s anthrax to learn whether staff at a worksite have been exposed to anthrax toxins and possibly provide further understanding of what factors might be associated with illness development following exposure. If staff have evidence of previous exposure, anthrax vaccination could be considered to prevent future infections of staff both at this worksite and other sites with similar risks.

In both investigations, no additional cases were identified despite numerous other individuals (including other welders) having similar occupational exposures or possible contact with contaminated clothing and gear (i.e., patient A’s household members). This suggests there may be a more complex array of risk factors beyond occupation and long-term welding exposures that predispose one to lung injury or infection [14,15]. Additional host factors such as iron overload and alcohol use disorder have been proposed as possible additional risk factors for welder’s anthrax [19].

To further reduce the risk of potentially fatal illnesses among welders and metalworkers, such as welder’s anthrax, several actions are recommended. Welding and metalworking employers should educate workers about the health hazards associated with welding, such as lung injury and infection, and measures that can minimize exposures to welding fumes, solvents, and other hazards. Employers should consider a workplace hazard assessment to assess the need for preventive measures, including the use of NIOSH-approved respirators as part of a written respiratory protection program and other preventive measures addressing higher levels of the hierarchy of controls [30]. Welding and metalworking employers, trade associations, and unions should consider targeted outreach to increase welders’ and metalworkers’ awareness about pulmonary infections, including welder’s anthrax, particularly among those in the U.S. Gulf Coast states where all known cases have occurred thus far.

Clinicians are urged to consider the possibility of infection with *B. cereus* group bacteria expressing anthrax toxin genes when treating patients with severe, rapidly progressive pneumonia or other anthrax-like diseases who have a history of working as a welder or other metalworker, particularly in the U.S. Gulf Coast states from Florida to Texas. Additionally, anthrax antitoxin may be indicated for patients with welder’s anthrax and is available through the Strategic National Stockpile in consultation with CDC [31].

Continued detection and surveillance of welder’s anthrax cases depend on close collaboration among clinicians and hospitals, local and regional health departments, the Laboratory Response Network, and CDC. As evidenced by the two cases in 2020, comprehensive epidemiologic investigations including a detailed work history and a robust environmental sampling plan are vital to further understand the source of exposure for these infections, relevant risk factors, and affected populations and geographic regions. Furthermore, worksite hazard assessments and application of the hierarchy of controls framework may help prevent illnesses such as welder’s anthrax.

## Figures and Tables

**Table 1:** Laboratory testing results for *B. cereus* group bacteria containing anthrax toxin genes from 185 environmental samples collected during the investigation of patient A’s source of exposure.

Number of Samples	Sampling Site *	Sampling Location	Sample Type ^†^	Real-Time PCR Result ^§^	Culture Result	WGS Result
1	Worksite	Front of oil tank access door	Soil	Positive	Positive	*B. tropicus* ^¶^
3	Worksite	Front of oil tank access door	Soil	Positive	Negative	N/A
4	Worksite	Worksite grounds	Gravel	Positive	Negative	N/A
2	Worksite	Grinder tools and cabinets	SS swab in NB	Positive	Negative	N/A
Total 10 real-time PCR-positive samples from worksite (8%)						
3	Residence	Work boots	MF swab in PBS	Positive	Negative	N/A
1	Residence	Work lunch cooler	SS swab in NB	Positive	Negative	N/A
Total 4 real-time PCR-positive samples from work-related clothing and gear at residence (22%)						
1	Worksite	Beneath oil tank ladder	Soil	Inconclusive	Negative	N/A
1	Worksite	Worksite grounds	Gravel	Inconclusive	Negative	N/A
1	Worksite	Rubber mats and wood pallets	SS swab in NB	Inconclusive	Negative	N/A
1	Worksite	Welding rods and container	SS swab in NB	Inconclusive	Negative	N/A
Total 4 real-time PCR-inconclusive samples from worksite (3%)						
55	Worksite	Various locations	Soil	Negative	Not done	N/A
11	Worksite	Various locations	Gravel	Negative	Not done	N/A
41	Worksite	Various locations	SS swab in NB	Negative	Not done	N/A
11	Worksite	Various locations	MF swab in PBS	Negative	Not done	N/A
Total 118 real-time PCR-negative samples from worksite (89%)						
2	Residence	Work-related clothing/gear	Filter cartridge	Negative	Not done	N/A
4	Residence	Work-related clothing/gear	SS swab in NB	Negative	Not done	N/A
8	Residence	Work-related clothing/gear	MF swab in PBS	Negative	Not done	N/A
Total 14 real-time PCR-negative samples from work-related clothing and gear at residence (78%)						
18	Residence	Yard—various locations	Soil	Negative	Not done	N/A
5	Residence	Yard—various locations	Gravel	Negative	Not done	N/A
12	Residence	Yard—various locations	SS swab in NB	Negative	Not done	N/A
Total 35 real-time PCR-negative samples from yard at residence (100%)						

Abbreviations: MF = macrofoam; N/A = not applicable; NB = neutralizing buffer; PBS = phosphate-buffered saline; SS = sponge-stick; WGS = whole genome sequencing. Shaded rows indicate subtotals of real-time PCR results by sampling location and percentage by sampling site. * The two sampling sites were patient A’s worksite in Louisiana (*n* = 132 samples) and residence in Mississippi (*n* = 18 samples from work-related clothing and gear; *n* = 35 samples from the yard). ^†^ Surface and subsurface soil and gravel samples were collected using sterile plastic spatulas and deposited into 50 mL conical polycarbonate screw-cap tubes. Soil and gravel samples were collected within a 2.5 cm × 2.5 cm square at a depth of up to 3 cm. Any reused plastic spatulas were cleaned with a 10% bleach solution between samples (which was preferred over pH-adjusted bleach for use in the field and deemed adequate for disinfection of environmental surfaces outside of a laboratory setting). Sterile sponge-stick swabs were soaked in 10 mL neutralizing buffer prior to swabbing and deposited into sterile Whirl-Pak^®^ plastic bags. Sponge-stick swabs were swabbed within 25 cm × 25 cm squares in overlapping “S”-patterns using all sides and edges of the sponge [20]. Sterile macrofoam swabs were soaked in neutralizing buffer prior to swabbing and deposited into sterile 15 mL conical polycarbonate screw-cap tubes. Macrofoam swabs were swabbed within 10 cm × 10 cm squares in overlapping “S”-patterns using all sides and edges of the sponge [20]. ^§^ A sample was considered real-time PCR-positive if two extractions yielded a positive result and was considered inconclusive if only one of two extractions yielded a positive result. *B. tropicus* is a member of the *B. cereus* group [12]. ^¶^ *B. tropicus* is a member of the *B. cereus* group.

**Table 2:** Single nucleotide polymorphism (SNP) distances between patient A’s clinical isolate, the environmental isolate from patient A’s worksite, and other isolates of *B. cereus* group bacteria containing anthrax toxin genes of multilocus sequence type 78 (ST-78).

	LA2020 Clinical	LA2020b Environmental	G9241 ^1^	03BB87 ^2^	LA2007 ^3^	BcFL2013 ^4^	BC-AK ^5^
**LA2020** **Clinical**	0						
**LA2020b** **Environmental**	2	0					
**G9241**	39	39	0				
**03BB87**	120	120	147	0			
**LA2007**	216	218	213	319	0		
**BcFL2013**	244	246	241	347	62	0	
**BC-AK**	1005	1005	1000	1113	1139	1165	0

Row and column headers in bold indicate various isolates of *B. cereus* group bacteria containing anthrax toxin genes of multilocus ST-78 [7,8,9,11]. *B. cereus* strain BC-AK, https://www.ncbi.nlm.nih.gov/biosample/SAMN06448760 (accessed on 26 May 2022).

## Data Availability

Sequences for the isolates are pending submission to a public repository and are available upon request. Patient data presented in this paper may be available upon request and have been published elsewhere.

## Appendix A

Detailed environmental sampling methodology for the investigation of patient A and patient B’s source of exposure to *B. cereus* group bacteria containing the anthrax toxin genes, and description of sampling sites.

Patient A—Worksite (Louisiana)

Surface and subsurface soil and gravel samples were collected using sterile plastic spatulas and deposited into 50 mL conical polycarbonate screw-cap tubes. Soil and gravel samples were collected within a 2.5 cm ×A 2.5 cm square at a depth of up to 3 cm. Any reused plastic spatulas were disinfected with a 10% bleach solution between samples (which was preferred over pH-adjusted bleach for use in the field and deemed adequate for disinfection of environmental surfaces outside of a laboratory setting). Sterile sponge-stick swabs were soaked in 10 mL neutralizing buffer prior to swabbing and deposited into sterile Whirl-Pak^®^ plastic bags. Sponge-stick swabs were swabbed within 25 cm × 25 cm squares in overlapping “S”-patterns using all sides and edges of the sponge [20]. Sterile macrofoam swabs were soaked in neutralizing buffer prior to swabbing and deposited into sterile 15 mL conical polycarbonate screw-cap tubes. Macrofoam swabs were swabbed within 10 cm × 10 cm squares in overlapping “S”-patterns using all sides and edges of the sponge [20].

The specific area within the worksite where patient A worked was outdoors and included a cylindrical oil tank with a radius of approximately 13 m. It was surrounded by gravel, soil, crushed shells, machinery, and two equipment trailers, and the worksite was contained within a rectangular perimeter of soil and vegetation measuring approximately 61 m × 76 m (Figure 1).

The sampling strategy incorporated professional judgment sampling, systematic sampling, and random sampling. The team divided the worksite into quadrants. Soil samples were collected around the perimeter spaced equally apart (*n* = 46; approximately 6 m apart), around the circumference of the oil tank (*n* = 6), and from two areas of interest where rainfall had collected, under the oil tank ladder (*n* = 4) and in front of the oil tank access door (*n* = 4). Gravel samples (*n* = 16) were collected from four randomly selected areas within each quadrant. Sponge-stick swabs were swabbed on the surface beneath the gravel (either wood pallets or rubber mats) (two in each quadrant; *n* = 8 total), the oil tank wall around the circumference of the tank at alternating heights (ground level, 1 m above ground, 2 m above ground; *n* = 14 total), the oil tank ladder steps (two from the bottom, middle, and top sections; *n* = 6 total), the top platform of the oil tank roof (*n* = 2), two machines (surface of a lift and the back and front of an air compressor; *n* = 3 total), and inside the two equipment trailers (welding masks, rods, and containers, and metal grinders and grinder cabinets; *n* = 12 total). Macrofoam swabs were swabbed on the oil tank ladder rungs (two from the bottom, middle, and top sections; *n* = 6 total) and crevices on the top platform of the oil tank roof (*n* = 5).

Patient A—Home and Yard (Mississippi)

Patient A also had multiple exposures to soil and dust around his residence, and work-related clothing and gear left at his residence were available for sampling. The sampling strategy incorporated professional judgment sampling and random sampling. Samples were collected as described above except that macrofoam swabs were soaked in phosphate-buffered saline (PBS) prior to swabbing instead of neutralizing buffer. This included 35 samples collected at sites of exposure to soil and dust around the property: sponge-stick swabs of the inside of a mobile home being used for storage (*n* = 5), lumber patient A had recently removed from the mobile home storage (*n* = 5), and a trailer used for horse feed (*n* = 2); and soil samples from beneath the lumber patient A removed (*n* = 2), next to a burn pit (*n* = 1), around a horse paddock (*n* = 5), around a tomato and vegetable garden patient A had recently planted (*n* = 10), and the gravel driveway (*n* = 5). An additional 18 samples were collected from work-related clothing and gear: macrofoam swabs of two pairs of work boots (top and inside treads from the right and left boot of each pair; *n* = 8 total) and a pair of laundered work jeans (*n* = 3); filter cartridges (*n* = 2); and sponge-stick swabs of a work lunch cooler (bottom and top/sides; *n* = 2 total) and the truck patient A used to travel to work where work-related clothing and gear were frequently present (driver’s side floorboard and pedals and passenger side floorboard; *n* = 3 total).

Patient B—Worksite (Harris County, Texas)

Patient B’s worksite, which manufactures proprietary fixtures for the oil and gas industry, had one office and two large fabrication shops surrounded by areas of concrete, grass, and packed gravel. One shop was predominantly for wood fabrication, and the other for metal fabrication. Both shops had large bay doors that usually stay open to allow increased airflow. His specific workstation was located next to a large bay door; outside the door was a grassy area, dirt field, and an area adjacent to the building set up for plasma cutting, which he did not perform. The area across from the woodworking shop was packed gravel and grass. Forklifts were used to move wood and metal around the worksite. The floors of both shops were concrete. There were no local exhaust systems in either shop; shop fans and wall-mounted exhaust fans were used for increased circulation.

Samples were collected following a similar protocol to the investigation related to patient A. The sampling strategy incorporated professional judgment sampling, systematic sampling, and random sampling. Broom bristles were collected by cutting off ~3-inch piece of bristle and inserting it into 50 mL conical polycarbonate screw-cap tubes. Environmental samples were collected from the worksite’s two fabrication shops and around the exterior of the worksite. Each fabrication shop was divided into a grid (*n* = 24 grids in the metal shop; *n* = 14 grids in the wood shop), with at least one sample selected from each area. A greater number of samples were collected in areas where the deceased individual was noted to have worked, including his workstation in the wood shop (*n* = 36 samples) and his locker in the metal shop (*n* = 2 samples). Materials that were routinely used to perform his work, personal PPE, and the individual’s bicycle (pedals and handlebars), which was used to travel to and from work, were sampled. Soil samples were collected along the perimeter of the entire worksite. A greater number of samples were collected in outdoor areas on the worksite property: around a picnic table underneath a tree where patient B ate lunch and took post-meal naps (*n* = 5 samples). Two additional areas were sampled that were adjacent to and on each side of the wood shop’s mostly open large bay door, which was immediately next to the patient’s indoor workstation: a gravel area where plasma cutting was conducted by other staff and a muddy/grassy area (*n* = 4 samples). A field with a mix of grass, dirt, and gravel across from the wood shop was divided into a grid with at least one sample selected from each area (*n* = 6 grids) (*n* = 7 samples).

## Appendix B

Worksite safety and home safety recommendations developed by anthrax and occupational safety and health subject matter experts for locations where *B. cereus* group bacteria containing anthrax toxin genes were detected.

**Intended Recipients**	**Rationale**	**Recommendations**
Worksite safety officer and welding employer	*B. tropicus* containing anthrax toxin genes was detected in a soil sample at patient A’s worksite, and it was a genetic match to the patient’s clinical isolate. Multiple other environmental samples tested real-time PCR-positive for the anthrax toxin genes. Therefore, the bacteria were still present at the worksite at the time of sampling and may pose a public health risk.	- Use a labor-management health and safety working group to discuss these recommendations and develop an action plan. Those involved in the work can best set priorities and assess the feasibility of the recommendations for the specific situation at this workplace. Helpful guidance can be found in “Recommended Practices for Safety and Health Programs”: https://www.osha.gov/shpguidelines/index.html. - Educate employees and contractors on hazards associated with welding and *Bacillus cereus* group bacteria expressing the anthrax toxin genes and train them on measures to help minimize potential exposures and recognizing signs and symptoms of inhalation anthrax-like illness. - Conduct a hazard assessment for all welders and welding supervisors at this worksite, given the apparent link between this bacterium and welders/metalworkers. Additional guidance on welding safety may be found in the OSHA Fact Sheet “Controlling Hazardous Fume and Gases during Welding”: https://www.osha.gov/Publications/OSHA_FS-3647_Welding.pdf. - Require all employees and contractors to always wash their hands before eating, drinking, or smoking, and each time they leave the workplace. - Do not allow employees to consume or store food or drink in work areas. ◦ Ensure that employees have adequate break areas—separated from work areas by closed doors—for eating, drinking, and storing food. - Avoid dry sweeping and avoid use of compressed air to clean dust, dirt, and other debris off work surfaces, tools, and equipment. ◦ Use a vacuum equipped with a HEPA filter or wet cleaning methods to clean surfaces. ◦ Provide, at a minimum, annual training to ensure compliance with approved cleaning practices. - Provide a dedicated space for employees and contractors to change in and out of their work clothing to reduce the risk of taking contaminants home. - Encourage employees and contractors with possible work-related health concerns to talk to their healthcare provider about their exposures at work. - Offer smoking cessation programs at no cost to employees and contractors. Encourage those who smoke to participate in smoking cessation programs. Smoking cessation may decrease respiratory symptoms worsened by workplace exposures.
Family members sharing residence with patient A	Multiple environmental samples of work-related clothing and gear at patient A’s residence tested real-time PCR-positive for the anthrax toxin genes. Given that *B. tropicus* containing anthrax toxin genes was detected in a sample at the worksite and was a genetic match to the patient’s clinical isolate, it is possible these items were contaminated with the same bacteria that caused patient A’s illness and may pose a public health risk.	Family members may choose to keep or discard patient A’s work-related clothing and gear. - If anyone is discarding any of these items, they should wear disposable gloves and place the items into a sealed plastic bag. The bag should be placed outside, preferably in a garbage bin, and thrown away with regular household garbage. After anyone handles the bag, they should remove their gloves by turning them inside out and dispose of them, and then wash their hands with soap and water [32]. If keeping any items, the following steps to safely clean them are recommended: - Work clothing: Wearing disposable gloves, place any unwashed clothing into a sealed plastic bag until the clothes can be placed directly into a washing machine. The clothing can be washed as normal using laundry detergent. - Work boots: Wearing disposable gloves, wash the boots with soap and water and remove any dirt or debris with a brush or sponge. Then spray the exterior of the boots with a 10% bleach solution * (one-part regular household bleach to nine-parts water) and let it sit for at least five minutes before wiping them clean. - Work lunch cooler and other items: Wearing disposable gloves, spray the exterior with a 10% bleach solution * (one-part regular household bleach to nine-parts water) and let it sit for at least five minutes before wiping it clean. - Hand hygiene: After anyone handles these items, they should remove their gloves by turning them inside out and dispose of them, and then wash their hands with soap and water [32]
* A 10% bleach solution was preferred over pH-adjusted bleach for safe use outside of a laboratory setting and was deemed adequate for disinfection of environmental surfaces. The resulting concentration of chlorine should be at least 5000 parts per million (ppm).		
